# Random support vector machine cluster analysis of resting-state fMRI in Alzheimer's disease

**DOI:** 10.1371/journal.pone.0194479

**Published:** 2018-03-23

**Authors:** Xia-an Bi, Qing Shu, Qi Sun, Qian Xu

**Affiliations:** College of Information Science and Engineering, Hunan Normal University, Changsha, P.R. China; Nathan S Kline Institute, UNITED STATES

## Abstract

Early diagnosis is critical for individuals with Alzheimer's disease (AD) in clinical practice because its progress is irreversible. In the existing literature, support vector machine (SVM) has always been applied to distinguish between AD and healthy controls (HC) based on neuroimaging data. But previous studies have only used a single SVM to classify AD and HC, and the accuracy is not very high and generally less than 90%. The method of random support vector machine cluster was proposed to classify AD and HC in this paper. From the Alzheimer's Disease Neuroimaging Initiative database, the subjects including 25 AD individuals and 35 HC individuals were obtained. The classification accuracy could reach to 94.44% in the results. Furthermore, the method could also be used for feature selection and the accuracy could be maintained at the level of 94.44%. In addition, we could also find out abnormal brain regions (inferior frontal gyrus, superior frontal gyrus, precentral gyrus and cingulate cortex). It is worth noting that the proposed random support vector machine cluster could be a new insight to help the diagnosis of AD.

## Introduction

Alzheimer's disease (AD) belongs to the central neurodegenerative disorders, which is featured with insidious onset and the progressive course of chronic [1]. The progress of AD is irreversible, however, early treatment with AD could alter the impact of the disease [2], such as slow or delay the disease process [3, 4]. Therefore, early diagnosis is critical in clinical practice. Magnetic Resonance Imaging (MRI) has been widely used to diagnose AD [5, 6]. As the resting-state functional magnetic resonance imaging (fMRI) is an often-used noninvasive technique for detecting brain activities and is more simple-economical [7], it has been widely employed to diagnose AD [8].

Machine learning is an effective tool, and able to extract information from functional magnetic resonance imaging (fMRI) data and predict pathology progression [9, 10]. In a number of machine learning methods, the classification method is particularly useful in AD pathology [11]. Support vector machine (SVM) is considered to be one of the best classification methods in machine learning [12]. Compared with other methods such as decision trees and Bayesian networks, SVM has obvious advantages such as the high accuracy [13], elegant mathematical tractability [14], and direct geometric interpretation [15]. In addition, SVM does not require a lot of training samples to avoid overfitting [16]. Therefore, SVM has drawn the attentions of researchers in the neuroimaging field and has been used to extract meaningful information from high-dimensional fMRI data [10, 17].

In recent years, support vector machine has been used in the study of disease classification. Jongkreangkrai et al. (2016) used the SVM to classify AD and HC based on the features of hippocampus and amygdaloid volume [18]. Zhan et al. (2015) applied multimodal support vector machine to identify the conversion from HC to MCI or AD by using MRI and positron emission tomography (PET) data, and the results showed that using multimodal data was significantly better than using a single modality [19]. As mentioned above, SVM is shown to be featured with high performance in classification. Beheshti et al. (2016) made SVM as a classifier and used the structural MRI data as features, and the classification accuracy of AD and HC was up to 92.48% [20]. Wottschel et al. (2015) employed support vector machine to predict the occurrence of clinical isolation syndrome (CIS) in the second clinical attack, and the SVM correctly predicted the presence (or the absence) in 71.4% of patients at 1 year, and in 68% at 3 years [21]. Valli et al. (2016) used structural MRI data as features of support vector machines for distinguishing patients who suffered from high risk of psychosis with accuracy of 72% (p <0.001) [22]. Retico et al. (2015) applied support vector machine and recursive feature elimination to identify AD based on whole grey matter, and the classification performance reached to AUC = (88.9±0.5)% in 20-fold cross-validation on the AD/HC dataset [11]. Zhang et al. (2015) regarded the discriminate regions that distinguished AD from HC as features of support vector machine based on 3 kinds of kernels among which the polynomial kernel showed the highest average accuracy of 92.36% [12]. By employing a stack automatic encoder and a feature representation based on deep learning, the accuracy of AD diagnosis reaches to 95.9% [23].

Previous studies have consistently used a single SVM to classify AD and HC, and the classification effect is often not ideal. The classification accuracy is not very high and generally less than 90%. In this paper, a random SVM cluster is proposed and used to distinguish AD from HC, and the accuracy is up to 94.44%. Although the accuracy is not the highest, it is relatively high. In addition, when the number of SVMs is 370, the accuracy of the random SVM cluster could be stabilized at 90%, which fully shows that the random SVM cluster has a good classification performance. It is worth mentioning that we could find out the optimal feature set to effectively distinguish between AD patients using and HC without all the features, and the accuracy could be as high as 94.44%. By employing the random SVM cluster, the abnormal brain regions such as inferior frontal gyrus (orbital part) could also be found out. Thus the random support vector machine cluster provides a new insight into the diagnosis of AD.

## Materials and methods

### Ethics statement

The ADNI study was approved by Institutional Review Board (IRB) of each participating site, these are the Banner Alzheimer’s Institute, Case Western Reserve University, Emory University, University of South Florida, Tampa, University of Rochester Medical Center, Washington University, St. Louis, Rhode Island Hospital, Georgetown University, Premiere Research Institute, Cleveland Clinic Lou Ruvo Center for Brain Health, University of Kentucky, Yale University School of Medicine, University of Alabama, Birmingham, Northwestern University, Ohio State University, Albany Medical College, St. Joseph's Health Center—Cognitive Neurology, Brigham and Women's Hospital, New York University Medical Center, Mount Sinai School of Medicine, Dent Neurologic Institute, McGill University / Jewish General Hospital Memory Clinic, University of California, San Francisco, Medical University of South Carolina, University of California, San Diego, University of Kansas, Oregon Health & Science University, Stanford University, University of California, Davis, University of Michigan, Ann Arbor, Wake Forest University Health Sciences, University of Texas, Southwestern MC, Rush University Medical Center, University of California, Los Angeles, University of Southern California, Johns Hopkins University, Indiana University, Banner Sun Health Research Institute, Parkwood Hospital, Boston University, Mayo Clinic, Jacksonville, Nathan Kline Inst. for Psychiatric Rsch, University of Pittsburgh, Duke University Medical Center, Baylor College of Medicine, Wien Center for Clinical Research, Dartmouth Medical Center, University of British Columiba, Clinic for AD & Related, Mayo Clinic, Rochester, University of California, Irvine, Sunnybrook Health Sciences Centre, Butler Hospital Memory and Aging Program, University of Wisconsin, University of Pennsylvania, University of California, Irvine (BIC), University of Iowa, Howard University, Columbia University. All ADNI subjects together with their legal representatives should have written informed consent before collecting clinical, genetic and imaging data.

### Subjects

Our experimental data was acquired from the Alzheimer's Disease Neuroimaging Initiative (ADNI) (http://adni.loni.usc.edu/) database, which includes AD individuals and HC. There are various neuroimaging data including fMRI images.

First of all, the selected data should ensure that each subject has resting-state fMRI data. Secondly, each subject should have mini-mental state examination (MMSE) scores and clinical dementia rating (CDR) scores to ensure that the data is homologous. Finally, 25 AD patients and 36 HC are selected out on the basis of the above criteria.

### Data acquisition

The 3.0-T Philips Medical Systems MRI scanner was used to acquire fMRI images. In the scanning process, the subjects were required to relax, do not think and lying in the scanner. Sequence parameters were as follows: pulse sequence = GR, TE = 30ms, TR = 3000ms, flip angle = 80degree, data matrix = 64*64, pixel spacing X = 3.31mm, pixel spacing Y = 3.31mm, slice thickness = 3.3mm, no slice gap, axial slices = 48, time points = 130.

### Data preprocessing

As the signal-to-noise ratio of the fMRI image is not high, the collected data need to be preprocessed to decrease the impact of noise on the functional magnetic resonance image. The Data Processing Assistant for Resting-State fMRI (DPARSF) software (http://d.rnet.co/DPABI/DPABI_V2.3_170105.zip) was employed to preprocess all collected resting-state fMRI data. For each subject, the whole preprocessing process is divided into 9 steps, details are as follows: converting DICOM format into NIFTI format; removing the first 10 time points; slicing timing; realigning; normalizing the functional images into the echo-planar imaging (EPI) template; smoothing by the full width half maximum (FWHM); removing the linear trend to eliminate the residual noise that systematically increases or decreases as time goes by [24]; temporal filtering to retain 0.01–0.08 Hz fluctuation; removing covariates to eliminate the impacts of physiological artifacts [25, 26], non-neuronal blood oxygen level dependent (BOLD) fluctuations and head motion [27].

### Sample feature of subjects

After completing a series of data preprocessing steps as mentioned above, we began to determine the sample feature. This paper used the functional connectivity as the sample feature, and the following is the process of the feature.

Firstly, the images were divided into 90 brain regions (45 in each hemisphere) in the light of the Automatic Anatomical Labeling (AAL). Then, the time series of each region are obtained. Next, the Pearson correlation coefficient of every two regions is calculated. Lastly, 4005 (90*89/2) functional connections could be obtained as the subsequent experimental feature.

### The random SVM cluster

In the neuroimaging, there are many features that can be extracted. For instance, the gray matter volume [28] and cortical thickness [29] can be extracted as features of structural MRI data, and the functional connectivity [30] can be extracted as features of fMRI data. Functional connectivity of the brain is generally extracted as the feature in the traditional application of SVM based on fMRI data. Thus each subject has 4005 functional connections, which is regarded as the high-dimensional feature [31]. In the existing literature, there is a default method to handle the high-dimensional feature, that is, reducing the redundant features and then building the model [32, 33]. There are many methods to remove redundant features, and each method has its own advantages and disadvantages [34]. It is undeniable that directly removing redundant features is a relative straightforward method and has the advantage of speeding up computation as well as improving performance, but it would also lead to the loss of some information to some extent [35].

In this paper, a new random support vector machine cluster is proposed to randomly select the samples and the features to establish multiple SVMs. The random SVM cluster is combined by these SVMs and used for classification and feature selection. This method achieves the purpose of dimension reduction to a certain extent and also has good classification performance.

#### The design and classification accuracy of the random SVM cluster

In the neuroimaging, the number of features is generally numerous. The traditional approach to dealing with the high-dimensional feature has some limitations, and thereby a new approach called random SVM cluster is proposed in this paper. The detailed process is as follows.

**Fig 1:**
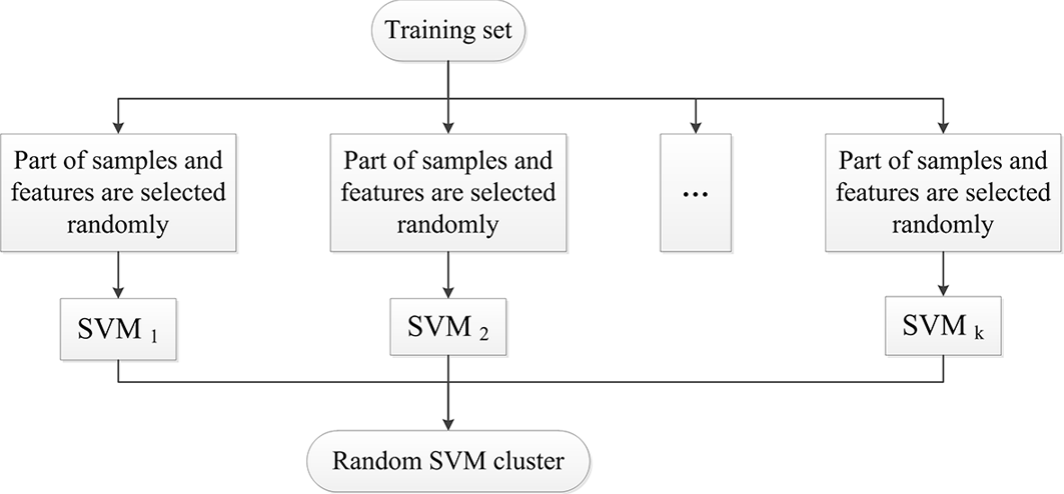
The design of the random SVM cluster.

Firstly, we randomly divide the sample set N into three components. They are the training set N1, the test set N2 and the validation set N3, where N = N1 + N2 + N3. In each time, n samples are randomly selected from the training set N1 and d dimensional feature is randomly selected from the 4005 dimensional sample feature. A single SVM is formed by the selected samples and features. The process is repeated for k times and k SVMs are finally formed which are used to construct a random SVM cluster. Fig 1 shows the design of the random SVM cluster. After constructing a random SVM cluster, we use test set N2 to optimize parameters.

Since each SVM is set up by selecting part of the features randomly, it is quite effective in dealing with high-dimensional feature data. Since samples and features of each SVM are selected randomly, each SVM is different. In other words, the SVMs are featured with diversity which makes the random SVM cluster have a good generalization performance.

When a new sample arrives, the k SVMs respectively categorize the new sample and then the majority of votes is used to predict the category of the new sample. Similarly, we predict the category of each sample in the validation set N3 based on the random SVM cluster. Then we compare the predicted category with the real category to judge whether they are consistent, and count the number of consistent situations which is denoted by Nc. The accuracy of the random SVM cluster equals to Nc/N3.

#### Extracting features from the random SVM cluster

As the selected features of each SVM are different, they have distinct performances. In this paper, the accuracy of SVM is used as a criterion to evaluate the quality of selected features. The features that make a significant contribution to the accuracy of SVM are called the "important features".

**Fig 2:**
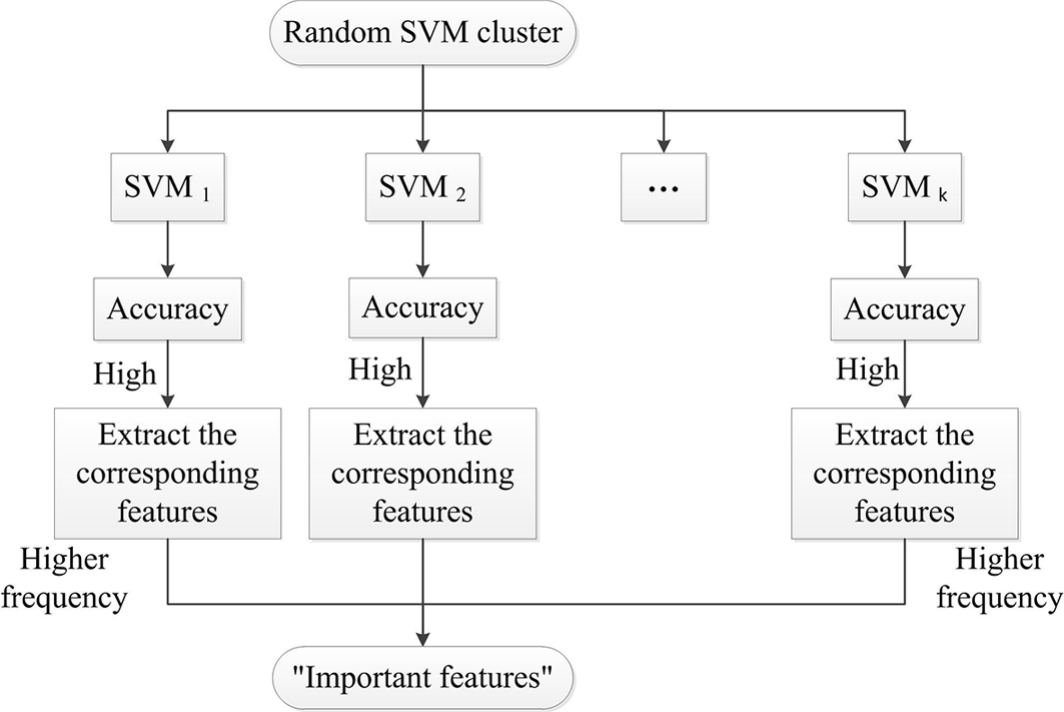
Selecting "important features".

The specific approach of selecting "important features" is as follows. Firstly, we predict the accuracy of each SVM in the random SVM cluster and sort their accuracy from the largest to the smallest. Secondly, we extract the features used in SVM with high accuracy to form a feature matrix. Lastly, the frequency of each feature in the feature matrix is counted and the features with the higher frequency are referred as the "important features”. Fig 2 shows the process of selecting "important features".

It is referred that the important features have great contributions to the accuracy of SVM, thus they also contributions to the accuracy of the random SVM cluster. As the important features distinguishingly contribute to the accuracy, we select features which are more important to the accuracy of the random SVM cluster from important features and these features are called the optimal feature set. The accuracy of the random SVM cluster is used as the criterion to find the optimal feature set.

#### The abnormal brain regions

As mentioned above, the optimal feature set has the greatest contribution to the accuracy of the random SVM cluster, so it could be regarded as the significant difference between AD and HC. In this paper, we make the optimal feature set as the abnormal functional connectivity. As functional connectivity is associated with two brain regions, the corresponding brain regions are the abnormal brain regions. We use the weight to denote the abnormal degree. The bigger the weight is, the more abnormal the brain region is. The weight of each region can be assessed by the number of regions associated with functional connections. In particularly, if there is no functional connection associated with the brain region, the weight of the brain region is 0.

### Experiment design

In this paper, the experiment is divided into five parts:

1. Building a random SVM cluster with 500 SVMs. 60 samples are divided into 40 training samples, 2 testing samples and 18 validation samples. The number of selected features and selected samples is $\sqrt{4005}\approx 62$ and 40 respectively. Each SVM uses Radial Basis Function (RBF) as kernel which is provided by the SVM toolbox (http://see.xidian.edu.cn/faculty/chzheng/bishe/indexfiles/indexl.htm).
2. Finding the optimal number of SVMs. In the above step, we subjectively make 500 as the number of SVMs, but it is not clear whether 500 is appropriate, which remains to be checked. Therefore, the aim of this step is to define the optimal number of SVMs. Firstly, we change the number of SVMs from 5 to 600, and then calculate the accuracy of the random SVM cluster with different number of SVMs. The number of SVMs corresponding to the random SVM cluster with the highest accuracy is optimal.
3. Selecting "important features". In this step, we make 0.75 as a criterion for high accuracy and select 400 features with higher frequency as "important features" which is used to find the optimal feature set. In addition, the "important features" are arranged in frequency from high to low.
4. Finding the optimal feature set. In the first step, we select 62 features from 4005 functional connections features to build the random SVM cluster. But in this step, we select 62 features from "important features" to build the random SVM cluster. In order to find the optimal number of "important features", we change the number from 70 to 400. In short, the number of selected features which are used to build the random SVM is always 62. However, the number of total features drops to 400 "important features" from the beginning of 4005 functional connections features. Then we calculate the accuracy of the random SVM cluster with different number of total features, and the number of total features corresponding to the random SVM cluster with the highest accuracy is optimal.
5. Finding the abnormal brain region. According to the optimal feature set, we could obtain the weight of each brain region.

## Results

### The basic characteristics of participants

After the preprocessing, the head moving parameters are needed to be checked. The data of one HC cannot be included in the subsequent analysis because the translation exceeded ±2mm or rotation surpassed ±2 degrees. The chi-square test is applied in assessing gender differences between AD group and HC group, and age differences are assessed by the two-sample *t* test. Table 1 shows the detail results. It is indicated that no obvious gender difference (p = 0.693) and age difference (p = 0.168) exists in these two groups.

**Table 1:** Basic characteristics of AD and HC.

Variables (Mean ± SD)	AD (n = 25)	HC (n = 35)	P value
Gender (M/F)	12/13	15/20	0.693
Age (years)	74.59±7.03	77.09±6.69	0.168

Abbreviations: AD, Alzheimer's disease; HC, healthy control

### The accuracy of the random SVM cluster

After the parameter adjustment of the test set, the related parameters are adjusted as follows: cost parameter equals to Inf and width parameter gamma of RBF equals to 3.

**Fig 3:**
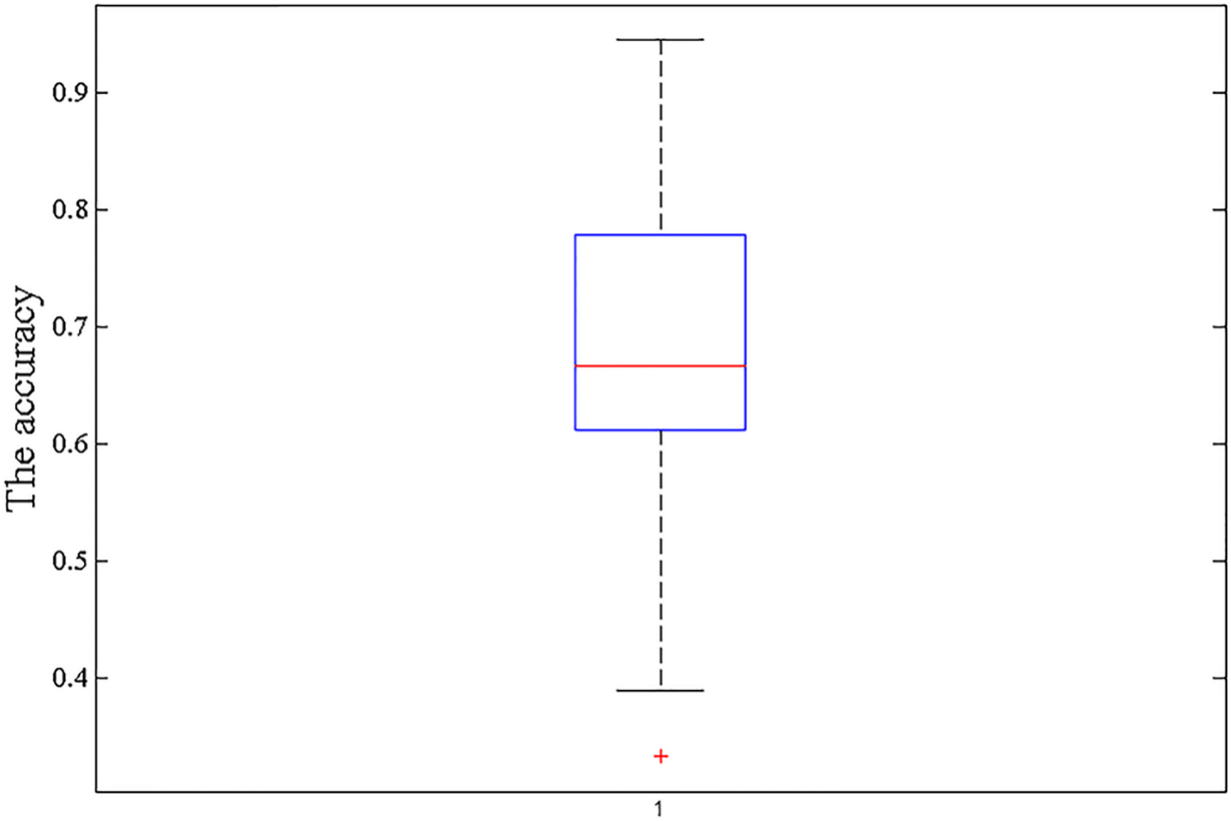
The accuracy of 500 SVMs.

As a random SVM cluster consists of many SVMs, we use 500 SVMs to build a random SVM cluster. As shown in Fig 3, about half of the single SVM's accuracy ranges from 0.6 to 0.78 and the average accuracy of these 500 SVMs is only about 0.65. Although the accuracy of a single SVM is not high, the accuracy of the random SVM cluster is as high as 94.44%. It is inferred that the random SVM cluster could compensate for the instability and low accuracy of a single classifier.

### The optimal number of SVMs and the optimal feature set

**Fig 4:**
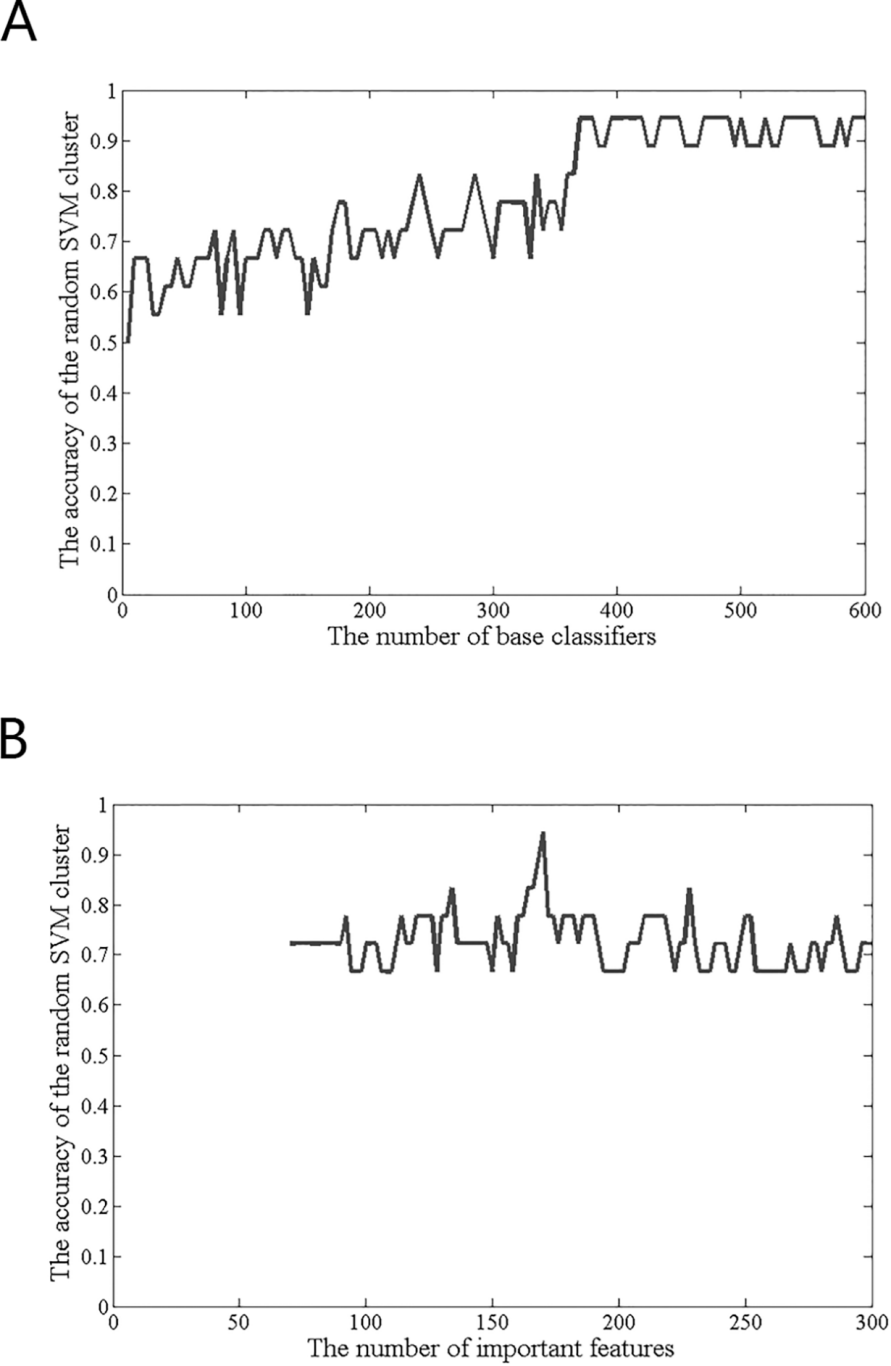
The optimal number of SVMs and optimal feature set.

Fig 4(A) shows the different levels of accuracy when the number of SVMs changes. As the number of SVMs rises to 370, the accuracy of the random SVM cluster begins to stabilize and fluctuates around the level of 90%. Therefore, 370 is regarded as the optimal number of SVMs in this paper, and the follow results are obtained on the 370 SVMs. Fig 4(B) shows the optimal feature set. When we make the first 170 features as the sample feature of the random SVM cluster, the accuracy of the random SVM cluster reaches to the highest and the accuracy is 94.44%. Therefore, the first 170 features are the optimal feature set.

### The abnormal brain regions

**Fig 5:**
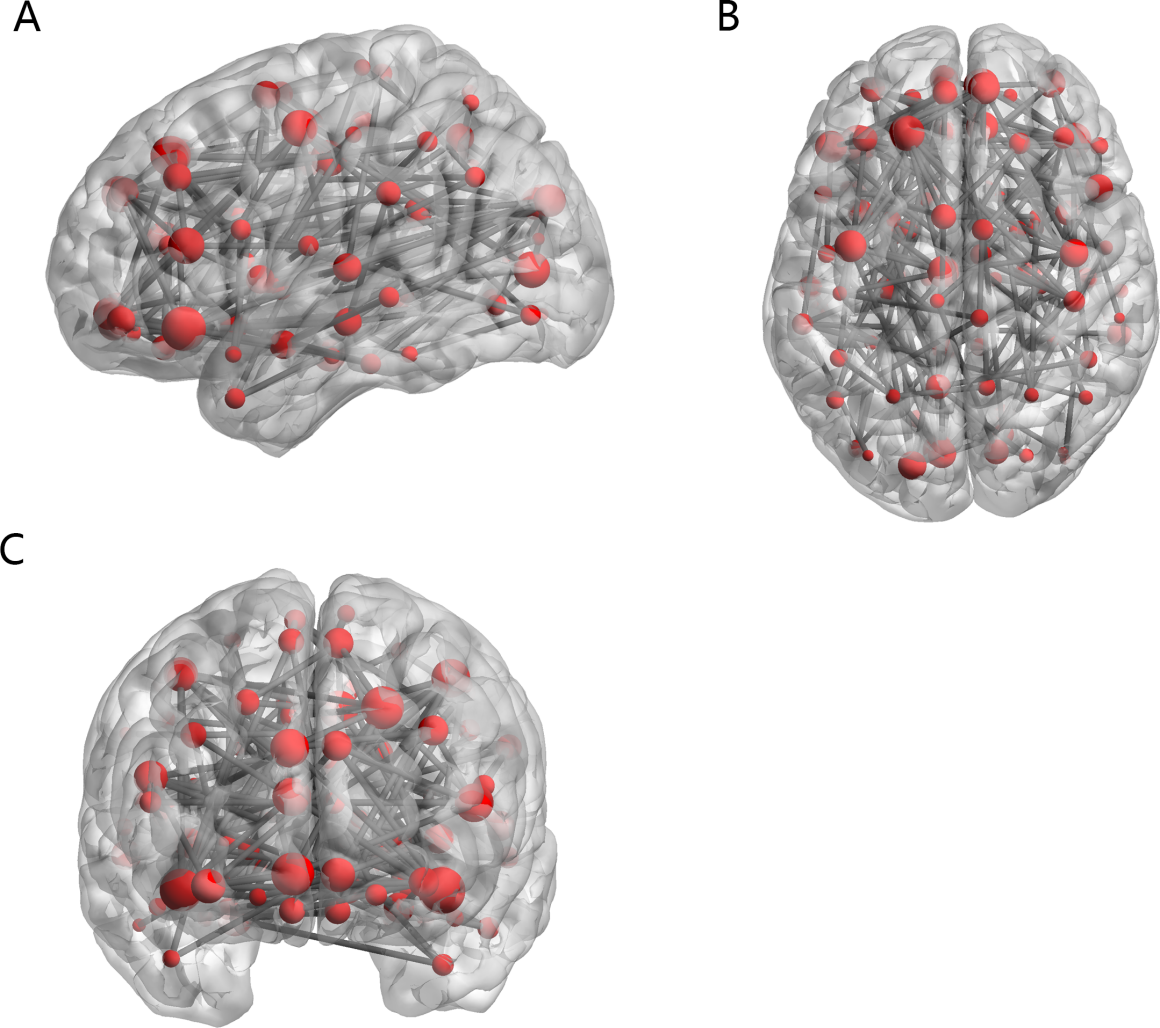
The distribution of 170 functional connections.

Fig 5 displays the distribution of 170 functional connections. The line represents the functional connectivity and the point represents the brain region. The sagittal, axial and coronal positions of the brain are respectively shown in Fig 5A, Fig 5B and Fig 5C.

**Fig 6:**
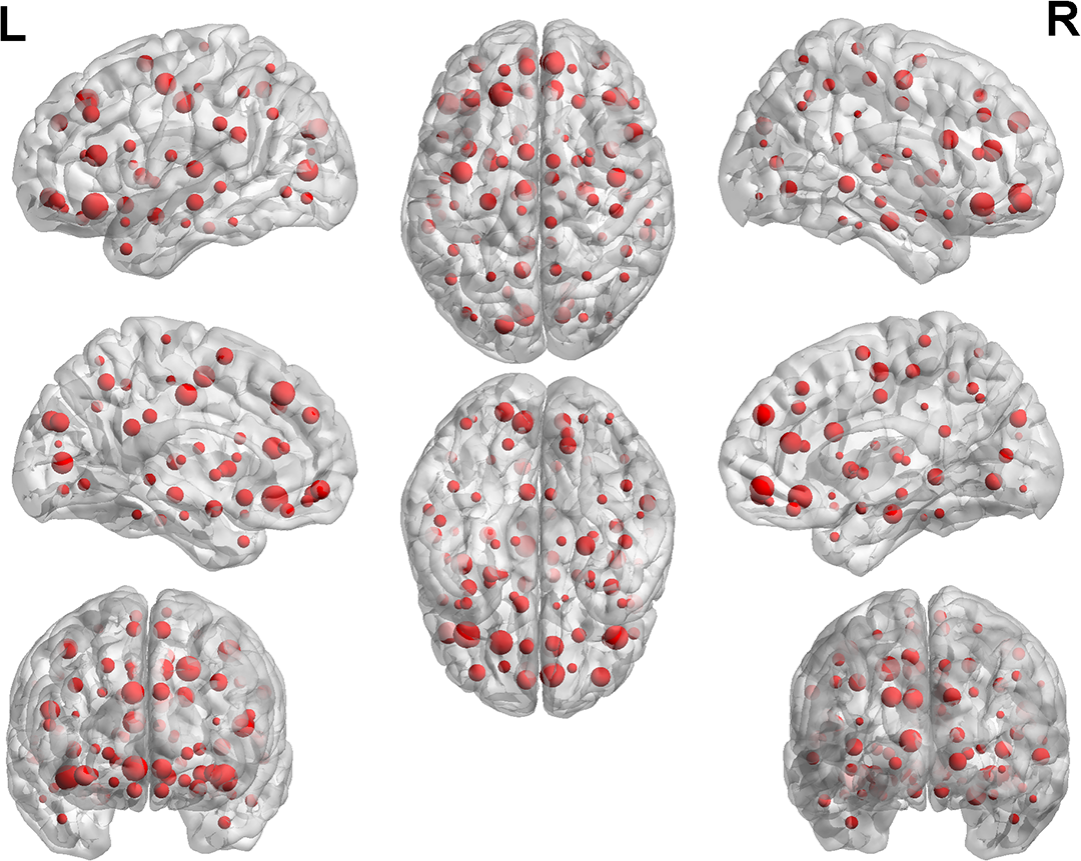
The weight of 90 brain regions.

**Fig 7:**
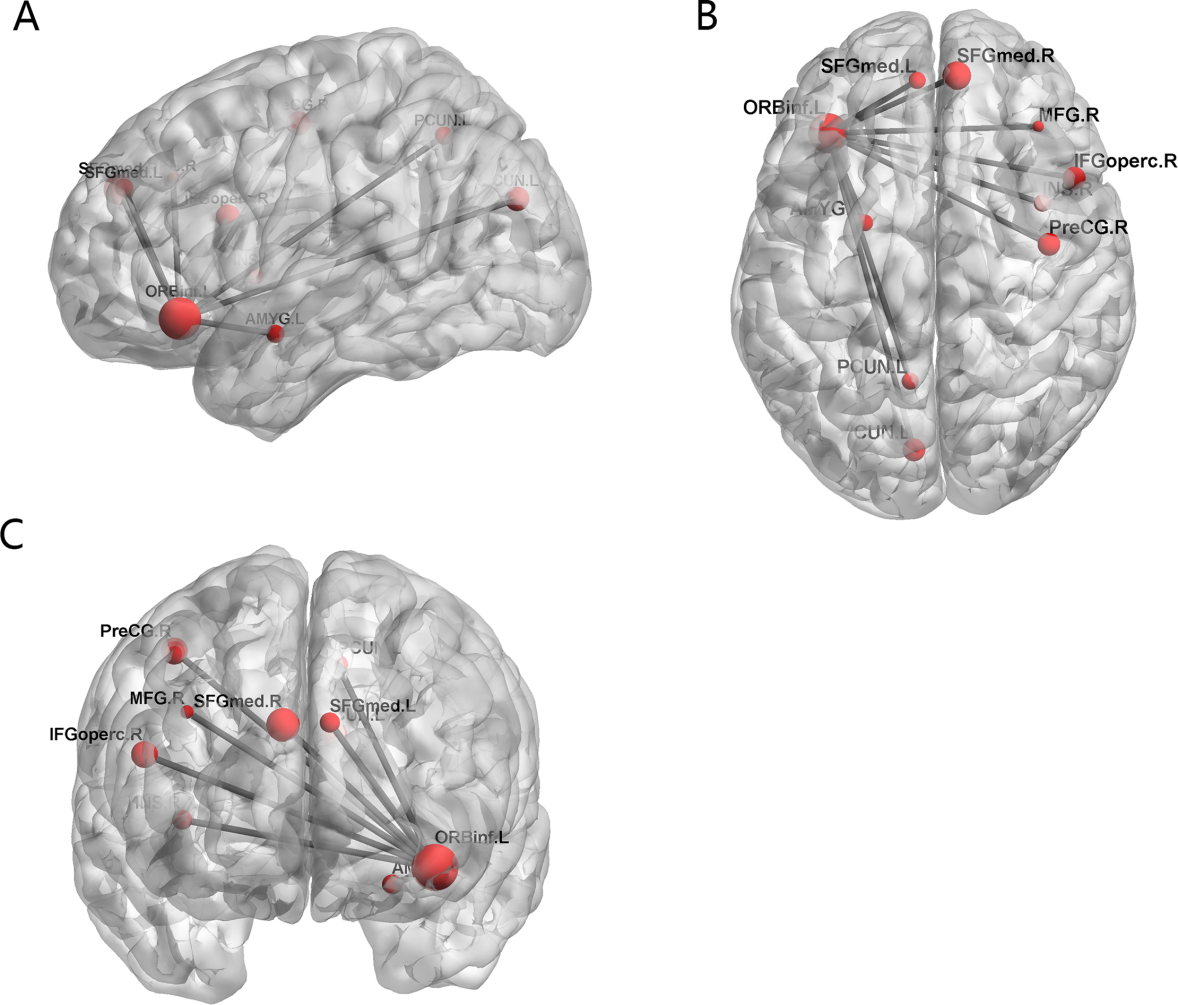
The functional connectivity between ORBinf.L and other brain regions.

The 170 functional connections help to find the abnormal brain regions. The weight of each region is obtained by the number of regions associated with the 170 functional connections. Fig 6 displays the weight of each brain region, and the size of the point represents the size of the brain weight. The specific weight values of some brain regions are shown in Table 2. The regions with the greater weight included the left orbital part of inferior frontal gyrus (ORBinf.L), the left superior frontal gyrus, dorsolateral (SFGdor.L), the right orbital part of inferior frontal gyrus (ORBinf.R), the right medial orbital of superior frontal gyrus (ORBsupmed.R), the left precentral gyrus (PreCG.L), the left triangular part of inferior frontal gyrus (IFGtriang.L), the right medial of superior frontal gyrus (SFGmed.R), the right anterior cingulate and paracingulate gyri (ACG.R), the left median cingulate and paracingulate gyri (DCG.L), the left calcarine fissure and surrounding cortex (CAL.L). We can learn from Table 2 that ORBinf.L has the largest weigh. Fig 7 shows the functional connectivity between ORBinf.L and other brain regions.

**Table 2:** The higher weight of brain regions.

Weight	Region
9	ORBinf.L
8	SFGdor.L ORBinf.R ORBsupmed.R
7	PreCG.L IFGtriang.L SFGmed.R ACG.R DCG.L CAL.L
6	PreCG.R ORBmid.R IFGoperc.R ORBsupmed.L PHG.R CUN.L SOG.L

## Discussion

### The performance of the random SVM cluster

In recent years, various predictive models have been employed to classify AD. Bayesian data mining with ensemble learning was used to distinguish AD and MCI and the accuracy was 81% [36]. Devanand et al. (2007) used the logistic regression model and the Cox proportional hazards model to identify mild cognitive impairment (MCI) and AD with accuracy of 83.3% [37]. Möller et al. (2015) used the gray matter density as feature and SVM as classifier to classify AD and HC, and the accuracy was 85% [38]. Chen et al. (2011) used large-scale network (LSN) analysis to classify AD and HC, and the accuracy was 87% [39]. Zhang et al. (2015) regarded the discriminate regions that distinguished AD from HC as features of support vector machine based on 3 kinds of kernels among which the polynomial kernel showed the highest average accuracy of 92.36% [12]. Beheshti et al. (2016) made SVM as a classifier and used the structural MRI data as features, and the classification accuracy of AD and HC was up to 92.48% [20]. By employing a stack automatic encoder and a feature representation based on deep learning, the accuracy of AD diagnosis reaches to 95.9% [23].

In this paper, a random SVM cluster is proposed and used to distinguish AD from HC, and the accuracy is up to 94.44%. Although the accuracy is not the highest, it is relatively high. In addition, when the number of SVMs is 370, the accuracy of the random SVM cluster could be stabilized at 90%, which fully shows that the random SVM cluster has a good classification performance. It is worth mentioning that we could find out the optimal feature set to effectively distinguish between AD patients using and HC without all the features, and the accuracy could be as high as 94.44%.

### Analysis of brain regions with greater weight

The results of this study show that the abnormal functional connectivity of AD compared with HC are mainly concentrated in frontal lobe and cingulate cortex. The frontal lobe and cingulate gyrus are the mainly regions of pathological changes in AD late stage [40, 41]. This shows that the abnormal brain regions which are found out by this study are consistent with the pathology progression of AD. Detailed analyses of the abnormal brain regions are conducted in the following.

(1) Orbitofrontal Cortex

In this study, we found that the weight of frontal gyrus (left orbital part) in all abnormal brain regions was the largest. It is indicated that the frontal gyrus contribute to classification of the random SVM cluster.

Orbitofrontal cortex and amygdala have long been considered to be a region which regulate changes in behavior, especially those that are guided by changes in the reward environment [42, 43]. Lesions of orbitofrontal cortex affect the reversal study in primates and rodents [44, 45]. Orbitofrontal cortex is part of the prefrontal cortex (PFC) which is important for flexible response to change environmental contingencies [46]. Thus, the orbital part may be involved in the prediction of the event and the past experience evaluation [47]. Rosenberg et al. (2015) pointed out that orbitofrontal cortex was associated with apathy in AD [48]. Nestor et al. (2015) provided evidence that orbitofrontal cortex gray matter had a unique contribution to intelligence [49].

Previous studies of Alzheimer's disease have concluded that the orbitofrontal cortex is abnormal. Woodward et al. (2015) found that orbitofrontal hypometabolism was greater in AD patients [50]. Cavedo et al. (2016) found that cortical thinning of left orbitofrontal cortex as well as anterior cingulate cortex (ACC) decreased significantly in AD patients [51]. Nakaaki et al. (2013) found that gray matter volume decreased in right ACC, posterior cingulate cortex (PCC) and orbitofrontal cortex in AD patients [52]. This fully demonstrates that the results of this paper are consistent with previous studies.

The abnormal connectivity of the orbitofrontal cortex with other brain regions is likely to lead to social cognitive degradation, memory loss, apathy, etc. in AD patients. Our experiments show that orbitofrontal cortex is significantly abnormal and the results provide a great help for the clinical diagnosis and treatment of Alzheimer's disease.

(2) Superior frontal gyrus (SFG)

In this study, we found that the weight of the superior frontal gyrus in the abnormal brain regions was relatively larger than others. It is manifested that the SFG contribute to classification of the random SVM cluster.

Hu et al. (2016) showed that activation of the right superior frontal gyrus was associated with more efficient reaction suppression and less motor urgency [53]. Changed functional connectivity and cortical thickness of the SFG was associated with impulsive responses in patients with posttraumatic stress disorders [54]. The right SFG has a specific role in actively controlling the impulsive responses [55, 56].

In the plentiful studies of AD, the superior frontal gyrus has been found to be abnormal. Zhang et al. (2015) detected 30 AD-related brain regions such as SFG, precentral gyrus, cingulate gyrus [12]. dos Santos Tascone et al. (2017) found that gray matter volume of the AD subgroup with few neuropsychiatric symptoms obviously reduced in the right SFG and the left ACC [57]. Wang et al. (2015) found that grey matter (GM) of AD group in right fusiform gyrus and right superior frontal gyrus decreased [58]. This demonstrates that our findings are coherent with existing literatures.

The abnormal connectivity of the superior frontal gyrus with other brain regions is likely to lead to impulsive responses in AD patients. This result contributes to the clinical diagnosis and treatment of AD.

(3) Precentral gyrus

In this study, we found that the weight of the precentral gyrus in the abnormal brain regions was relatively larger than others. This shows that the precentral gyrus also plays a critical role in the classification of the random SVM cluster.

Existing literature points out that precentral gyrus activation may be associated with the motor demands [59]. The activation of left precentral gyrus reflects the sensory motor control of the mouth, tongue and pharynx in expressing language activities [60]. Recently, a meta-analysis of cerebral activations exposed that the left precentral gyrus was more activated when reading nonwords compared with words during single word reading in fluent adult readers [61].

Existing literature of Alzheimer's disease has pointed out that the precentral gyrus is abnormal. Zhang et al. (2015) proposed a new method to detect 30 AD-related brain regions such as anterior cingulate, precentral gyrus [12]. Wang et al. (2016) detected 17 regions related to AD such as postcentral gyrus, precentral gyrus and cingulate gyrus by using the pure computer-vision technique [62]. Nishida et al. (2015) found that beta 2 connectivity slightly declined between right parahippocampal gyrus and right precentral gyrus in AD patients [63]. Guo et al. (2016) showed that the connectivity between the precentral gyrus to the middle cingulum and supplementary motor area decreased in AD patients with depression compared with non-depressed AD [64]. This illustrates that the results of this study are coherent with existing literatures.

The abnormal connectivity of the precentral gyrus with other brain regions may lead to language impairment in AD patients. This result could improve the clinical diagnosis and treatment of Alzheimer's disease.

(4) Cingulate cortex

We found that the weight of the cingulate cortex in the abnormal brain regions was comparatively larger than others in this study. This shows that the cingulate cortex plays a decisive role in the classification of the random SVM cluster.

The anterior cingulate cortex (ACC) could have an essential role in pain processing and pain-gating at the cortical level [65]. The anterior cingulate cortex (ACC) participates in regulating cognitive and emotional behavior. If the ACC is damaged, it may result in personality changes, impulses, and social behavior impairment [66].

In the abundant studies of Alzheimer's disease, researchers have discovered that the cingulate cortex is abnormal. Landin-Romero et al. (2017) found that the PCC cortex in AD patients has larger atrophy than behavioural-variant frontotemporal frontotemporal dementia (bvFTD) individuals [67]. Huey et al. (2017) found that posterior cingulate cortex had atrophy in AD patients and this brain area was also independent of apathy [68]. dos Santos Tascone et al. (2017) found that gray matter volume of the AD subgroup with few neuropsychiatric symptoms obviously reduced in the right SFG and the left ACC [57]. Wang et al. (2013) found that compared to HC, the structural interaction between right inferior parietal cortex and PCC increased in the AD patients [69]. This displays that the results of this study are coherent with existing literatures.

The cingulate cortex abnormalities can be seen as the mark of Alzheimer’s disease and it is probably to cause cognitive impairment in AD patients. This result could promote the clinical diagnosis and treatment of AD.

Our study contributes to introducing the random SVM cluster method to analyze fMRI data of AD and HC. This model has a good classification performance and its classification accuracy reaches to 94.44%. However, it also has several limitations. Firstly, the functional connectivity used in the study was established at the brain level, and future studies could use functional connectivity at voxel level. Secondly, although this study could have an excellent result only by using the functional connectivity as features, in the future studies we could improve the random SVM cluster classification performance by integrating the functional connectivity and other imaging modality measurements. Finally, in order to recognize AD earlier, it is suggested to detect early stages of AD such as mild cognitive impairment, as well as predicting future cognitive decline from functional connectivity measures in future studies.

## Supporting information

S1 Table The feature of 60 participants in the experiment. (XLSX)
